# Study of Caspase 8 Inhibition for the Management of Alzheimer’s Disease: A Molecular Docking and Dynamics Simulation

**DOI:** 10.3390/molecules25092071

**Published:** 2020-04-29

**Authors:** Syed Sayeed Ahmad, Meetali Sinha, Khurshid Ahmad, Mohammad Khalid, Inho Choi

**Affiliations:** 1 Department of Medical Biotechnology, Yeungnam University, Gyeongsan 38541, Korea; sayeedahmad4@gmail.com (S.S.A.); ahmadkhursheed2008@gmail.com (K.A.); 2 Department of Bioengineering, Integral University, Lucknow 226026, India; meetali.info@gmail.com; 3 College of Pharmacy, Department of Pharmacognosy, Prince Sattam Bin Abdul Aziz University, Alkharj 16278, Riyadh, Saudi Arabia; drkhalid8811@gmail.com

**Keywords:** Alzheimer’s disease, caspase 8, molecular dynamics, RMSD, RMSF

## Abstract

Alzheimer’s disease (AD) is the most common type of dementia and usually manifests as diminished episodic memory and cognitive functions. Caspases are crucial mediators of neuronal death in a number of neurodegenerative diseases, and caspase 8 is considered a major therapeutic target in the context of AD. In the present study, we performed a virtual screening of 200 natural compounds by molecular docking with respect to their abilities to bind with caspase 8. Among them, rutaecarpine was found to have the highest (negative) binding energy (−6.5 kcal/mol) and was further subjected to molecular dynamics (MD) simulation analysis. Caspase 8 was determined to interact with rutaecarpine through five amino acid residues, specifically Thr337, Lys353, Val354, Phe355, and Phe356, and two hydrogen bonds (ligand: H35-A: LYS353:O and A:PHE355: N-ligand: N5). Furthermore, a 50 ns MD simulation was conducted to optimize the interaction, to predict complex flexibility, and to investigate the stability of the caspase 8–rutaecarpine complex, which appeared to be quite stable. The obtained results propose that rutaecarpine could be a lead compound that bears remarkable anti-Alzheimer’s potential against caspase 8.

## 1. Introduction

The prevalence of Alzheimer’s disease (AD) continues to increase in parallel with aging, but no remedies have yet been developed that delay or inhibit AD-induced neurodegeneration [1]. The available evidence shows that AD is the result of the combined effects of environmental, genomic, epigenomic, and metabolic variables [2]. AD is the most common form of dementia and causes deficiencies in language and visuospatial skills, which are frequently accompanied by behavioral issues such as aggressiveness, apathy, and depression. It has been reported that genetics contributes around 70% to the risk of AD [3]. Furthermore, it has been estimated approximately 47 million people live with dementia, and it is predicted that this figure will more than treble by 2050. The prevention of AD needs a timely diagnosis and multidisciplinary management [4]. The formation of extracellular β-amyloid (Aβ) plaque aggregates and intracellular neurofibrillary tangles associated with the hyperphosphorylation of τ-protein in the cortical and limbic portions of the human brain underlie the pathogenesis of AD [4,5,6]. The disease also increases the activities of acetylcholinesterases, which contribute to cholinergic system function, and AD-associated dementia is known to involve extreme devastation of and complications in the cholinergic system [7].

Caspase activation is a significant step in the apoptotic pathway and prompts the proteolytic cleavage of proteins in neurons. Previously, it was believed that the classical hallmarks of AD, i.e., plaques and tangles, occur individually and do not involve caspase activation. However, recent findings indicate that tangles, plaques, and caspase activation contribute in concert to the pathogenesis of AD, and caspase-cleaved tau may initiate or promote the formation of tau tangles [8]. Caspase 8 is a large molecule known to participate in AD that is prompted by Aβ_1–40_ to induce apoptosis. Caspase 8 shows a significant role in causing AD by cleaving amyloid precursor proteins during apoptosis, leading to the increased formation of the amyloid-beta peptide [9]. Qian et al. (2015) suggested caspase 8 inhibition might suppress neuronal apoptosis and AD-associated movement impairments [10]. 

Natural neuroprotective compounds have been shown to provide promising outcomes when used to treat neurodegenerative diseases like AD and to have negligible side effects [11]. Rutaecarpine is a component of the alkaloid extract of the Chinese medicine Evodia, which has been reported to improve myocardial ischemia-reperfusion injury. Yan et al. (2013) suggested rutaecarpine has neuroprotective effects in cerebral ischemia-reperfusion injury and might recover neurological functions [12]. Thus, the present study was undertaken to evaluate the ability of rutaecarpine to inhibit caspase 8 by a molecular docking study and MD simulation with the aim of identifying a potential therapeutic approach for the treatment of AD.

## 2. Results

In the present study, we retrieved a list of natural compounds from the ZINC database (https://zinc.docking.org/) using filters such as “natural product”, “non-fda”, and “in-stock”, and these were subsequently filtered with the Lipinski rule of five; a total of 200 compounds were selected for molecular docking studies against caspase 8, and it was found that rutaecarpine (ZINC898237) bound most strongly with caspase 8, which was further validated by MD simulations. Our findings indicate that the neuroprotective effects of rutaecarpine require further study.

**Figure 1:**
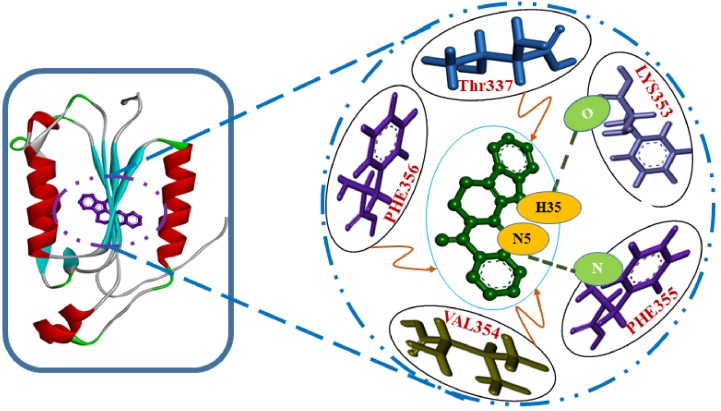
Lowest-energy docked structure of the caspase 8/rutaecarpine complex. Hydrogen bonds are represented by bold dashed green lines.

The docking studies showed human caspase 8 interacts with rutaecarpine through five amino acid residues, namely Thr337, Lys353, Val354, Phe355, and Phe356 (Figure 1) with a binding energy and inhibition constant of −6.13 kcal/mole and 75.68 μmol, respectively.

**Figure 2:**
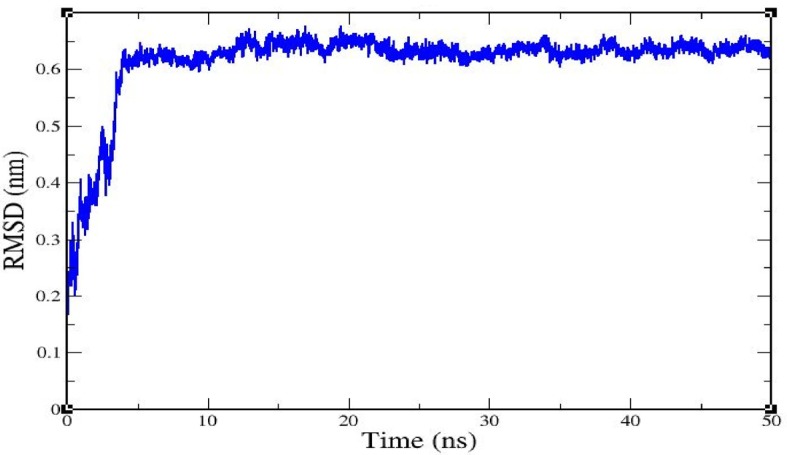
The root mean square deviation of caspase 8/rutaecarpine as determined by molecular dynamics (MD) simulation.

A root mean square deviation (RMSD) graph was obtained by MD simulation for the Cα backbone of the caspase 8–rutaecarpine complex, and it showed that the complex was stable. The complex reached equilibrium during the initial phase of simulation and then remained stable over 50 ns. The RMSD value of the complex gradually increased for 5 ns and then stabilized between 10–50 ns, as displayed in Figure 2.

**Figure 3:**
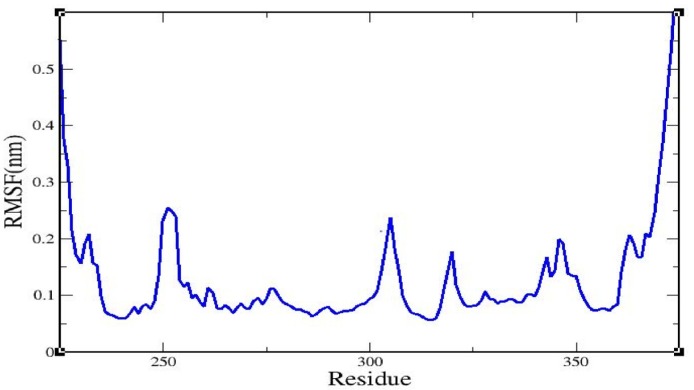
The root mean square fluctuation of the caspase 8/rutaecarpine complex.

Root mean square fluctuation (RMSF) values for all residues in the caspase 8–rutaecarpine complex were examined (Figure 3). 

**Figure 4:**
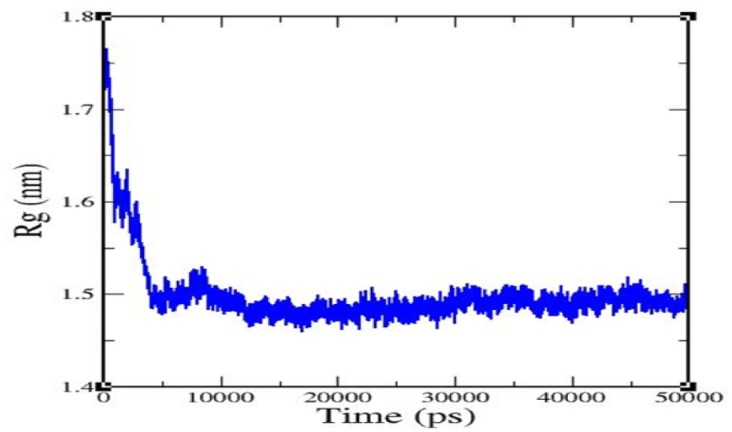
Radius of gyration of rutaecarpine against caspase 8.

The radius of gyration (Rg) value of the complex backbone was determined for a 50 ns trajectory (Figure 4), and it was found that the caspase 8/rutaecarpine complex was stable and densely packed.

The potential energy of the caspase 8–rutaecarpine complex was analyzed for 500 ps (Supplementary Figure S1A). The potential energy of complex stabilization was found to be approximately −1.2304035 × 10^6^ kcal/mol. The temperature of the system rapidly reached 300 K and then remained stable over the rest of the simulation time. The average temperature of the system was 299.638 K (Supplementary Figure S1B). The pressure fluctuated widely over the 100-ps equilibration phase. The average pressure was 10.1745 bar as shown in Supplementary Figure S1C, and the average density was 981.465 kg m^−3^. Density values were very stable over time, indicating that the system was well-equilibrated with respect to pressure and density (Supplementary Figure S1D).

**Figure 5:**
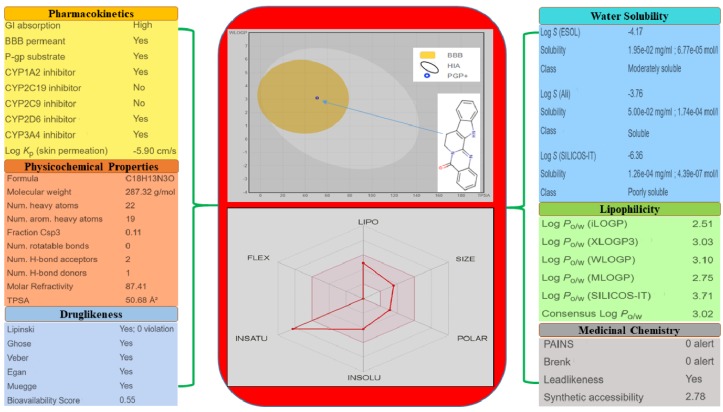
Physicochemical and drug-likeness properties of rutaecarpine.

SwissADME, a web tool, is used to predict the ADMET properties of the molecules [13]. The different drug-likeness properties of rutaecarpine, i.e., lipophilicity, water-solubility, pharmacokinetics, and physicochemical properties, are demonstrated in Figure 5. These predictions demonstrate that rutaecarpine lies in the safe region to be considered as a potential lead compound.

## 3. Discussion

Numerous preclinical/clinical studies have shown natural products could make valuable contributions to the treatment of AD [14]. Rutaecarpine was the first natural alkaloid used to treat cerebral ischemia-reperfusion injury and has been suggested for the treatment of vascular dementia [15,16]. Furthermore, molecular docking has been widely used to predict the bindings of small molecules and drugs to their protein targets and to predict their binding affinities and activities [17]. 

MD simulation is used to obtain binding mode information between receptors and ligands [18]. Here, we used huperzine A and quercetin as control molecules to validate receptor–ligand interactions. In previous studies, huperzine A appeared to have positive effects on the improvement of cognitive function and curing of AD [19], and quercetin showed significant protective effects on neuronal cells in AD [20,21]. The free energies of human caspase 8 binding with huperzine A and quercetin have been reported as −4.28 kcal/mol and −4.89 kcal/mol, respectively [22].

In the present study, two amino acid residues, LYS353 and PHE355, of caspase 8, that is, ligand: H35-A: LYS353:O and A:PHE355: N-ligand: N5 were found to be involved in H-bond formation with rutaecarpine, with H-bond distances of 1.60045 and 3.09333 Å, respectively. The donor, H35 of rutaecarpine, interacted with the O of LYS353, whereas the nitrogen of PHE355 interacted with acceptor N5 of rutaecarpine. It has been reported that hydrogen bonds formed among ligands and receptors more often contribute to the stability of the protein–ligand complex [23,24].

One amino acid residue of caspase 8 (Val354) was found to interact hydrophobically with rutaecarpine, whereas Phe355 was involved in Pi–Pi and cation–Pi interactions. Van der Waals, hydrogen bonding, and desolvation energy components contributed −6.44 kcal/mol to rutaecarpine and caspase 8 binding, and the electrostatic contribution was −0.04 kcal/mol. The total interacting surface area of the caspase 8–rutaecarpine complex was 485.76 Å^2^. The docked complex was subjected to MD simulation to confirm the stability of the rutaecarpine-to-caspase 8 interaction because the technique is known to usefully simulate bench-based experimental results [25,26]. The time-dependences of the MD trajectories were examined using the RMSD of complex and Rg of all backbone atoms [27]. RMSF is an important parameter that yields data about the structural adaptability of Cα atoms of every residue in the system [28]. The Rg is used to assess the overall dimensions and stabilities of the enzyme-ligand complex and is a function of the mass-weighted RMS distances of atoms from the center of mass [29]. The BOILED-Egg plot generated by SwissADME graphically represents rutaecarpine in Figure 5. It can be effectively concluded from the outcome that rutaecarpine crosses the blood–brain barrier (BBB) (yellow region). Daina and Zoete (2016) reported that ponatinib accurately lies inside the white ellipse but inside the BOILED-Egg′s yolk, too. This agrees with experimental data suggesting that ponatinib crosses the BBB [30,31]. 

## 4. Materials and Methods

### 4.1. Preparation of the Receptor Structure

**Figure 6:**
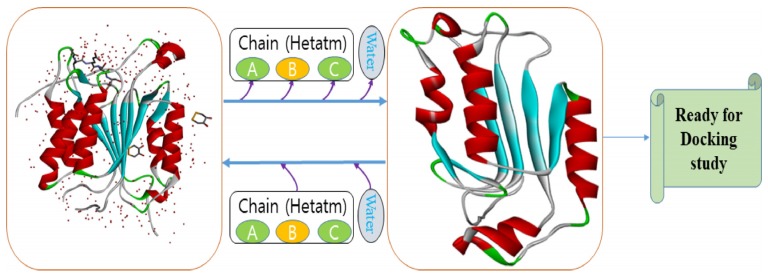
3D structure preparation of human caspase 8 for molecular docking analysis.

The three-dimensional (3D) structure of human caspase 8 (PDB ID: 1qtn) was retrieved from the protein data bank (PDB) [32] (Figure 6). A PDB file was prepared and heteroatoms were evacuated manually. 

### 4.2. Preparation of Ligand Structure

**Figure 7:**
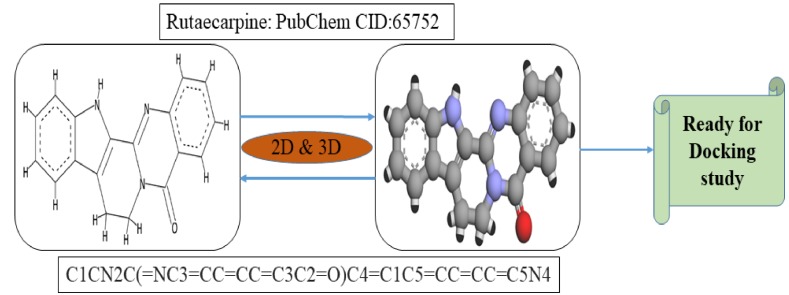
2D and 3D representations of the rutaecarpine molecule.

The simplified molecular-input line-entry specification notation of rutaecarpine (ZINC898237) was acquired from the PubChem database (PubChem CID: 65752). CORINA was used online to obtain the three-dimensional structures of rutaecarpine (Figure 7).

### 4.3. Molecular Docking Study

Rutaecarpine was subjected to molecular docking analysis with caspase 8 using the “Autodock 4.2” standalone molecular docking suite [33]. Gasteiger charges types were assigned for the compound, and the MMFF94 force field was applied for energy minimization. Non-polar hydrogen atoms and rotatable bonds were defined, and the docking estimations were performed on protein models. Hydrogen atoms, Kollman charges, and solvation factors were incorporated by the AutoDock tools. A box of 60 × 60 × 60 Å was formed using the Autogrid program. Docking simulations were accomplished by Lamarckian genetic calculations. The docking experiment involved 50 unique runs over 2,500,000 energy evaluations, with other default parameters. The best run coordinates of the compound and enzymes were visualized using Discovery Studio Visualizer.

### 4.4. BOILED-Egg Plot

The simplified molecular-input line-entry specification notation was entered into the online Swiss ADME web tool for the prediction of gastrointestinal absorption and brain penetration. These predictions were done utilizing the BOILED-Egg (Brain or Intestinal Estimated permeation) method. This method is principally founded on two parameters: (1) the lipophilicity of the compounds, assessed from the partition-coefficient (P) by a LogP value determined by the Wildman–Crippen method (WLogP); and (2) their polarity, dictated by a calculated topological polar surface area (tPSA) value [30]. It can be easily studied on the basis of the yellow-shaded yolk representing the physiochemical space for highly probable BBB penetration and the white space representing the physiochemical space for HIA absorption. Another parameter associated with this study is the P-gp active efflux pump as it transports the lipophilic drug out of the brain capillary endothelial cells that form the BBB [34].

### 4.5. MD Simulation Study 

MD simulation was performed using GROMACS 5.1.4 to analyze the stability and flexibility of the caspase 8–rutaecarpine complex [35]. The ProDRG server was used to prepare rutaecarpine topology files [36]. The complex was solvated in a cubic box of volume 377.187 nm^3^. The steepest descent algorithm for 50,000 steps with a cut-off value of 1000 kJ·mol^−1^ was applied for energy minimization. In addition, the LINCS algorithm [37] was used to constrain the bond lengths. NVT (constant number of particles, volume, and temperature) and NPT (constant number of particles, pressure, and temperature) phases of the equilibration were executed for 100 ps. Temperature coupling with a V-rescale, which is a modified Berendsen-thermostat, was done for an immersion with a temperature 300 K and a time constant of 0.1 ps, and pressure coupling was done with a Berendsen bath [36] with a time constant 2.0 ps. MD simulation was conducted for 50 ns. The outcomes such as the RMSD, RMSF, and Rg of the caspase 8–rutaecarpine complex were analyzed according to the time-dependent behaviors of MD trajectories.

## 5. Conclusions

In this study, the binding between the natural compound rutaecarpine and human caspase 8 was studied using a combination of molecular docking and molecular dynamics methods. Hydrogen bonds and hydrophobic interactions were found to play important roles in caspase 8–rutaecarpine complex formation. The binding energy and inhibition constant of the caspase 8–rutaecarpine complex were found to be −6.13 kcal/mol and 75.68 μmol, respectively. In addition, simulation analysis was performed to better understand backbone fluctuations and complex stability using RMSD and RMSF values. The results showed that the complex was stable over the simulation time. It is hoped our findings will be found useful by researchers searching for novel lead compounds for the management of AD.

## Supplementary Materials

Figure S1A: Potential energy analysis; Figure S1B: Temperature analysis; Figure S1C: Pressure analysis; Figure S1D: Density analysis.

## Abbreviations

AD	Alzheimer’s disease;
BBB	Blood brain barrier;
MD	Molecular dynamics;
RMSD	Root mean square deviation;
RMSF	The root mean square fluctuation;
Rg	The radius of gyration.
