# Supplementary figures and images for: Study of Caspase 8 Inhibition for the Management of Alzheimer’s Disease: A Molecular Docking and Dynamics Simulation
Source: Molecules. 2020 Apr 29;25(9):2071. doi: 10.3390/molecules25092071 (PMC7249184; doi:10.3390/molecules25092071)

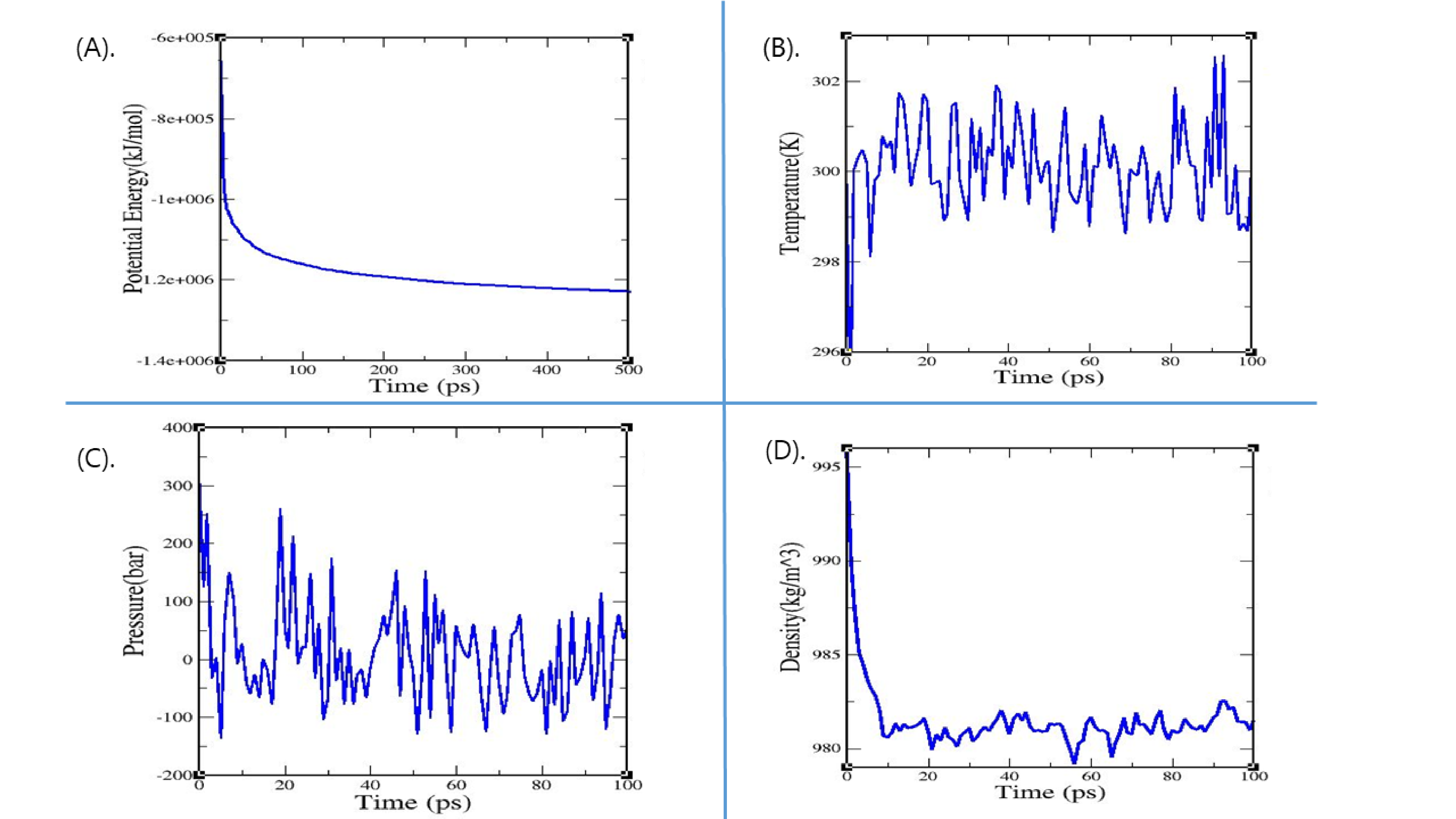

Supplement: Supplementary file 1 [file molecules-25-02071-s001.zip › molecules-793954-supplementary.tif]
